# Domiciliary Hospitalization through Wearable Biomonitoring Patches: Recent Advances, Technical Challenges, and the Relation to Covid-19

**DOI:** 10.3390/s20236835

**Published:** 2020-11-29

**Authors:** André F. Silva, Mahmoud Tavakoli

**Affiliations:** Institute of Systems and Robotics, Department of Electrical Engineering, University of Coimbra, 3030-290 Coimbra, Portugal; andre.fsilva@isr.uc.pt

**Keywords:** IoMT, Covid-19, wearable biomonitoring patches, stretchable circuits, fabrication, liquid metals

## Abstract

This article reviews recent advances and existing challenges for the application of wearable bioelectronics for patient monitoring and domiciliary hospitalization. More specifically, we focus on technical challenges and solutions for the implementation of wearable and conformal bioelectronics for long-term patient biomonitoring and discuss their application on the Internet of medical things (IoMT). We first discuss the general architecture of IoMT systems for domiciliary hospitalization and the three layers of the system, including the sensing, communication, and application layers. In regard to the sensing layer, we focus on current trends, recent advances, and challenges in the implementation of stretchable patches. This includes fabrication strategies and solutions for energy storage and energy harvesting, such as printed batteries and supercapacitors. As a case study, we discuss the application of IoMT for domiciliary hospitalization of COVID 19 patients. This can be used as a strategy to reduce the pressure on the healthcare system, as it allows continuous patient monitoring and reduced physical presence in the hospital, and at the same time enables the collection of large data for posterior analysis. Finally, based on the previous works in the field, we recommend a conceptual IoMT design for wearable monitoring of COVID 19 patients.

## 1. Introduction

### 1.1. Background

The healthcare sector is rapidly changing. Many countries have implemented or are implementing value-based healthcare. The European Union supports the shift toward digital healthcare and a patient-centric approach through several funding programs [1]. Digital Healthcare and Domiciliary hospitalization are some important components of future healthcare. This is due to several reasons. First, hospital beds are scarce [2] and costly. Second, the world is getting older [3]. In 2025 the world is expected to have twice elderly as it had in 2010 [4]. Third, For those living in isolated zones, daily access to healthcare is challenging [5]. Chronic diseases are one example of medical monitoring at a distance; the current demographic dictates the number of people who are fighting with chronic diseases is increasing [6]. Quality of life is sensible and could increase, regarding the physical aspect, for people living with these conditions if they are monitored 24 h per day. The national health expenditures (NHE) of the United States of America are expected to almost double from 2012 to 2024 [7]. This situation can pose a strain on the country’s economy for the near future and the lack of access to health for part of the population [8].

Domiciliary hospitalization is a model of hospitalization in which the patient receives hospital-level care while staying at home. One important tool for a wider application of this hospitalization model is wireless patient monitoring. In the last decade, rapid advances were made on architectures for the Internet of things (IoT) and the Internet of medical things (IoMT), including compact and low consumption processing and communication chips. In addition, emerging technologies for the implementation of wearable patches have rapidly evolved. This includes technologies in printed and stretchable electronics and e-textile, printed sensors, including, as well, printed batteries, energy harvesting and supercapacitors. IoMT is expected to have a market of $322.2 billion by 2025, growing 29.9% from 2019 to 2020 [9]. Still, the Internet of medical things corresponds to only 7% of the Internet of things devices [10].

The Internet of things is the amalgamation of devices including sensors, actuators and software, all together constitute a network that exchanges data over the Internet. The Internet of Things concept was first discussed more than 20 years ago [11], but recent years observed an increasing interest in research and commercial investment in this field, for different applications [12,13], and increased from 19 billion devices in 2013 to 40 billion in 2019 [12]. The Internet of medical things (IoMT) is an area within the IoT that directly focuses on medical applications and deals with the acquisition, processing, transmission and storage of medical information through the amalgamation of specific devices (things) built to ensure patient safety and data security [14]. IoMT builds over a general IoT system. Different design concepts have been shown both using existent protocols, as well as commonly used bands, i.e., industrial, scientific and medical (ISM) bands [15], which includes both Bluetooth and ZigBee. Additionally, novel and emerging protocols for wireless sensor body network (WSBN) are being investigated. Some of the protocols were also implemented specifically for medical use, such as the medical implant communications service (MICS). The acceptance of the IoMT system among the population is generally more challenging than other IoT systems, as it deals with highly sensitive and personal data. Due to the sensitivity of these types of data, security and patient privacy are two important factors in IoMT design. On the sensing layer, as these devices are in direct contact with the patient body, it is desired to have systems that are soft, and stretchable, biocompatible, electrically safe, and comfortable for long-term use. In addition, as these are generally mobile devices, special attention is given to low-energy devices in order to enable long-time monitoring without the hassle of battery recharging.

### 1.2. The Relationship to the Global Epidemic

An example of domiciliary hospitalization was seen during the Covid-19 pandemic. Hospitals struggled with resources to fight the spreading of the virus [16]. Soon, in most countries, it was decided that most patients should be hospitalized in their homes, while in some countries, they received medical attention through daily phone calls [17]. However, making the decision of which patients to admit to the hospital was largely based on how the patient feels and not based on clinical measurements. During global epidemics, identifying causes and monitoring infected patients are important in order to support clinical decisions. Collection of large data can contribute, as well, to predict and prevent the spreading of the disease [18]. The use of such predictive models was already shown in past epidemics [19].

In addition, applying artificial intelligence (AI) algorithms to the obtained data can result in the discovery of important digital biomarkers, health indicators for the diagnostic or prediction of diseases, and quantification of health conditions. Algorithms for data-mining in healthcare are described in detail in [20] and include swarm intelligence, linear discriminant analysis, k-means clustering, decision tree, naïve bayes, vector quantization, artificial neural networks and finally fuzzy logic. A recent review article [21] also discussed AI extensively in IoMT.

Recently, adaptions and new approaches to deal with the Covid-19 started to emerge, since new inventions to help with disinfecting hands [22]. Solutions to diagnose without professional clinical assistance [23], using pooling sample diagnosis for a faster diagnosis [24] and general rapid large-scale testing [25]. Or the use of digital monitoring using smartphones, gathering big samples of data and using machine–learning algorithms [26]. However, some problems with large amounts of data also emerged. For example, the government applications for monitoring brought big security concerns and can be troublesome to implement on a large scale due to its reputations [27]. The Covid-19 poses many problems, an important one being the need for monitoring symptoms of infected patients in a reliable way without the need to resort to hospital resources.

That being said, there is a real need for a fast implementation solution of a reliable IoMT system, specifically tailored for the needs of each situation. The Covid-19 epidemic is a perfect example of this need. A specific IoMT system would be a good help for monitoring non-risk patients at home while relieving the stress of the national healthcare systems around the world. Additionally, the amount of data collected from this system would greatly help to study and fighting the current and future outbreaks, having in mind that the way the data are collected does not raise so many concerns about privacy and security. Even more, an IoMT system tailored for Covid-19 would monitor the well-being of the patients in their comfort at home without risking any new contaminations. Since it would be a constant monitorization, the system would also be able to react automatically in cases of emergency.

### 1.3. Domiciliary Hospitalization through Wearable Biomonitoring Patches: Article Overview

This article reviews recent advances and existing challenges for the application of wearable bioelectronics for patient monitoring. More specifically, we focus on technical challenges, and solutions, for the fabrication and application of IoMT solutions that are skin-conformal and comfortable.

In Section 2, we discuss the wearable bioelectronics possibilities for an IoMT system, namely, the possible skin interfacing bio-signals to monitor, in Section 2.1, and the IoMT general structure for patient monitoring, in Section 2.2, including a discussion of the technologies used for efficient and low-power wireless communications (section Technologies for Wireless Communications). This includes a range of technologies, including well-established general communication protocols, to more recent and emerging communication methods, such as fat inter-body communication.

We follow by discussing scientific, technical challenges and recent advances in conformal bioelectronics in Section 3. We introduce and discuss the recent trends and challenges regarding the materials and fabrication methods in Section 3.1, including wearable biomonitoring, printed and stretchable electronics and emerging technologies such as electronic tattoos. Then, we discuss the problem of energy supply and solutions that are currently being investigated in this domain, including printed and stretchable batteries, supercapacitors, and thin-film energy harvesting. The problem of power consumption is also discussed in Section 3.2.

Finally, Section 4 is focused on the discussion of a system to monitor Covid-19 patients. The essential parameters to monitor and how to do this monitoring, regarding types of conformal sensors are explored (Section 4.1)—ending the discussion with a proposed design for multisensor electrophysiological monitoring for a dedicated IoMT system for Covid-19 monitoring, in Section 4.2, including a prototyping solution and comparison on other current approach trends to the same problem.

In recent years, the wearable bioelectronics research field has been very active. Consequently, there exist some good review works on the subject. Namely, focusing on the epidermal, ocular and oral wearable biosensors [28]; describing wearable, flexible hybrid electronics (WFHE) [29]; or broad reviews of the parameters, mechanisms, materials and fabrications and performances and challenges [30]. We differentiate our work in the sense that we try to take a specific approach to some thematics, like the challenges faced for e-skin patches. Fabrication, long-term use and interfaced are explored, an essential review of energy solutions for the implementation is discussed, comparing batteries, several energy harvesting solutions and supercapacitors. While giving some insight as well, on the context, these sensors are inserted as complex systems. More specifically, we introduce the thematic of wireless communications and networks, explore body sensor networks and the broader concept of IoMT, where we intend to insert the wearable patch sensors. In the end, based on this approach, we explore the case of the Covid-19 epidemic and introduce the concept of building a fast prototype based on an IoMT system that would allow the domiciliary hospitalization of Covid-19 patients. This idea of rapid prototyping adaption could also be used in response to other emergencies.

## 2. Wearable Bioelectronics for an IoMT System

The first part of wearable bioelectronics is body-interfacing devices that acquire and transmit the electrophysiological data. These devices are generally composed of electronic components for acquisition, digital conversion, processing, and communication of data. Wearable biomonitoring devices are a category of wireless monitoring, which in addition to electronics and signal processing, includes research on materials and fabrication techniques that provide conformable and comfortable wearable monitoring [31,32,33]. Traditional wearables, such as smartwatches and fitness trackers, are widely used wearable monitoring devices. Nevertheless, these are generally limited both in terms of the location that can be applied (e.g., wrist or chest) and the type of signal that they can monitor. For instance, as these devices cannot be applied over the throat, they are not able to monitor throat activity. Soft and stretchable biomonitoring stickers do not have these limitations. They can be applied anywhere on the body, and as they conform better to the human soft tissues, they can provide a better signal, less prone to noise and human motion [34]. Other examples of futuristic patches are “electronic tattoos”, that are ultrathin films populated with microelectronics that are transferred to the human skin, similar to the temporary decorative tattoos. This category of devices is further explained in Section 3.1.

In this section, an overview of the parameters that can be measured by wearable bioelectronics systems will be presented. Next, the general structural design for an IoMT system will be presented, including sensing, communication, and application layers.

### 2.1. Skin Interfacing Electrophysiological Sensing: What Can Be Measured?

The human body emits various types of signals that contain important information about human health. These signals can be divided into various categories, based on the type of signal and the transducers that are necessary to measure them. The most common types of these signals include bio-electric, bio-Impedance, bio-acoustics, and bio-optical signals, mechanical and motion sensing, temperature sensing, and electrochemical measurements for sweat monitoring.

Bio-potential signals are parameters based on an electric signal produced by the body. This includes biopotentials that are generated by muscles, heart, and brain, i.e., electrocardiogram (ECG) for monitoring of heart, electroencephalogram (EEG) for brain signal acquisition, and electromyography (EMG) to measure the muscular activity.

Bioimpedance is the measurement of the impedance of a specific part of the body. This is usually measured as the response of the body upon the introduction of a small current [35]. Examples of an application include smart scales that measure and report body fat and body mass index. Other than body composition information, this can be as well used to measure heart pulse and other vital signs as blood flow or breathing rate [36]. Another example of an application is to monitor the skin impedance changes to analyze the galvanic skin response (GSR) as an indicator of human emotions. As a response to emotional responses, sweat glands activity increase, changing the conductance of the skin [37].

Bioacoustics are sounds produced by organs or other body components. This includes heart valve sounds, blood flow, lung inflation sounds and bowel movement [38,39]. Bioacoustics can be used, as well, to monitor the throat sounds. This can be used to monitor the throat vibration during talking [40], eating (to monitor daily food intake) [41], and coughs [42]. The application of the latter is under study for the possible classification of Covid-19 patients [43]. Some implementations also allow bio-acoustic sounds to be used as a gesture controller [44].

Bio-optical signals are produced using a light source or body imaging. This includes pulse oximetry to measure the blood oxygen and heart rate, using an LED and a photodiode that monitor changes on the light reflection resulting from the oxyhemoglobin concentration in the blood [45,46]. Another example of application includes using the optical spectra of the skin to study cancer cells [47].

Body temperature constitutes one important health indicator and can be measured with various techniques using an optical signal, using thermal cameras [48] or by contact sensors, including digital thermometers and thermistors [49].

Mechanical movements of the body can be used as well in monitoring the daily activity in general or in the analysis of motion and vibration of a specific part of the body. This is generally performed using accelerometers and gyroscopes [50]. Mechanical sensors can also be exploited for measuring muscular movements through force myography as an alternative to electromyography [51].

Other types of bio-signals are emerging as well. Chemical and electrochemical signals are the measure of the chemical composition of bio-fluids [52], such as sweat. Sweat analysis can be done with wearable and non-invasive methods and allow for easy tracking of bio-markers in their composition for the detection of genetic conditions and other kinds of diseases [53]. For more information on types of bioelectronics signals for biomonitoring, or HMIs, one can consult related review articles [54,55].

**Figure 1:**
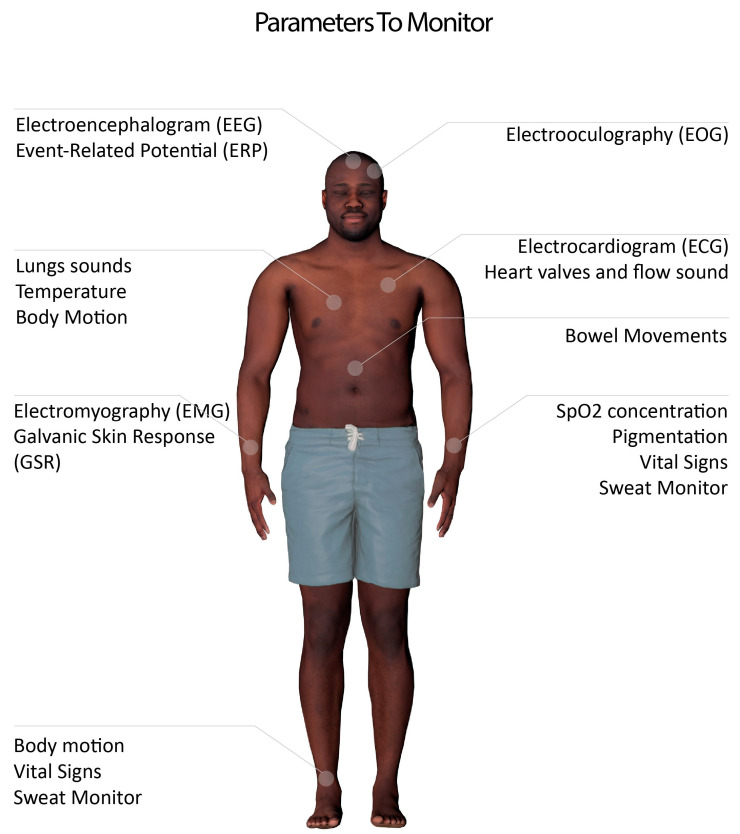
Parameters that can be monitored using an Internet of medical things (IoMT) system and wearable biomonitoring.

Figure 1, and Table 1, summarizes the main electrophysiological parameters that can be monitored by non-invasive bioelectronics devices.

### 2.2. IoMT System for Patient Monitoring and Domiciliary Hospitalization

Over the last years, different types of IoMT architectures were proposed and investigated [13]. These are generally multilayer systems that intend to assure safe data transmission and communication. Recent implementations generally propose three main layers, a wearable sensing layer, an intermediary data acquisition and transmission layer and a cloud computing layer [56,57]. The first two layers are sometimes combined, but the objective of the intermediary layer is to reduce the necessary components over the sensing layer and build a robust middle layer that is more flexible in data transmission to the cloud. With those implementations in mind, we discuss a simple general design for an IoMT system that can be adapted for specific uses.

**Figure 2:**
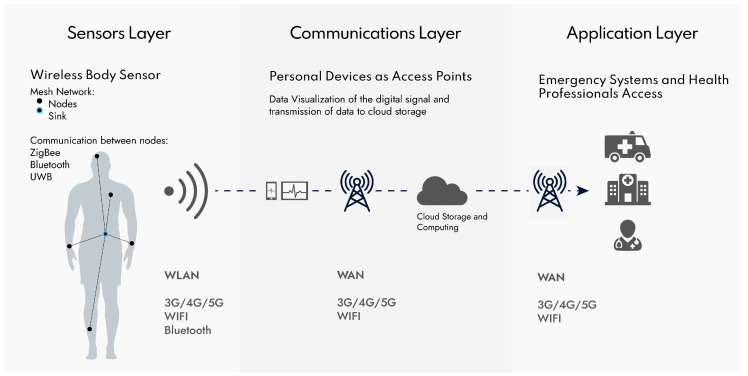
General implementation of an IoMT system for wireless patient monitoring and domiciliary hospitalization.

Figure 2 demonstrates the general structural design of an IoMT system for wireless patient monitoring and domiciliary hospitalization. Every implementation of an IoMT will vary; however, we can generalize three layers that help understand the general direction of the information:

First layer (sensors)—when several different sensors are implemented at various locations of the body, it is common to build a wireless sensor body network (WSBN) or wireless sensor network (WSN) as well. WSBN is defined as a network of various sensors connected to each other. Usually, in a mesh arrangement, even though other typologies are possible [15]. Sensors can be implantable, wearable and mobile (hold in hands or inside pockets). The sensors’ communications have a low range (~2 m) and have low power needs. Active research tries to improve the efficiency of the protocols to consume the least possible energy [58]. The band used for this low power network is mostly the common industrial, scientific and medical (ISM) band, or may be more specific, such as medical implants communications service (MICS) band. Both may be used for direct communication of a single sensory device, to an external device, or in a WSBN architecture. These bands allow for common technologies as Zigbee or Bluetooth to be used, but also some new approaches like fat–intrabody communication. The sensors layer was given that name for simplicity since some actuators are also possible to implement. Most of the challenges of this layer, though, belong to the sensors themselves. We will discuss wearable sensors challenges in Section 3. The mesh nodes from the WSBN connect to a central master, also called the sink. The master controls the communications with the sensors and communicates with an exterior gateway to the wireless local area network (WLAN) [59]. Energy and security of the data transmitted are of great importance.

Second layer (communications)—The master of the WSBN will communicate to the WLAN via common technologies like mobile communications, WIFI or Bluetooth. The power requirements of these types of networks are still important; however, not as limited as the sensors layer. In this case, it is characterized by the existence of a local gateway that can be a personal mobile device like a smartphone or tablet. The IoMT system gets easier and faster to implement, without the need for unnecessary gateway components. It can also be an ad-hoc device, specially built for this purpose. The mid-layer gateway can communicate both with the sensing layer and with the exterior network WAN, for instance, through 5G, WIFI or GPR. This serves as an intermediate layer for data storage and communication. Ideally, the sensor layer components are not always transmitting information, only being awake from time-to-time to transmit the fundamental information. For this reason, the data that arrives at the gateway is not yet treated a first raw part of information can be displayed and treated in the phone. However, after transmitting the data to the cloud server, it has fewer limitations in terms of computing power and energy, so it stores the data and can also be used to process the data applying algorithms of data-mining. The cloud server is located at the border with the application layer.

Third layer (applications)—As the cloud server manages the communications to the gateway system, it also handles data processing and data application. This way, it also needs to handle data access to health professionals or the patient through a website or smartphone application, as well as implement ambulatory emergency services to request medical attention in a domiciliary hospitalization system. For this reason, the application layer connects via WAN to personal devices as to other services like the ambulatory or other hospital storage center service. The biggest concern for the application layer is the security of the data. As it manages communications with different terminals, it is more vulnerable to exposition. Even though difficult, security is being researched with new approaches dealing with blockchain for a secure and decentralized implementation of the IoMT [60].

#### Technologies for Wireless Communications

Bluetooth was created to replace RS-232 cable connections with a wireless alternative. As such, continuous communication by Bluetooth requires 1 W of power [61] and is capable of 1–3 Mbit/s data communication over 500 m is possible (100 m advisable) [62]. Bluetooth low energy (BLE), originally from Nokia in 2006, was later integrated with Bluetooth 4.0 release in 2009 to address the necessity of very low power communication for IoT devices. The data transfer was slightly affected (1 Mbit/s data rate), but the power consumption was significantly reduced (0.05–0.1 W). This tremendous increase makes the BLE low energy, an excellent candidate for the use in IoMT devices. The newer version of Bluetooth is the 5; it was created with IoT applications in mind, thus allowing the use of Bluetooth low energy while promising increasing speed and range [63].

ZigBee was created with the objective of a low power alternative for implementation in home automation personal area network (PAN) while being less expensive. Zigbee has a slow data rate (250 kbit/s) but is very power efficient (consuming as low as 9.3 mA while in working mode) [64]. It uses mostly the bands of 2.4 GHz (915 MHz and 868 MHz for different zones) with mesh typology. Implementing a Zigbee network is low cost and power-efficient, becoming an excellent alternative for WSBN implementation, plus it allows for theoretical 65,000 devices connected in the same network. ZigBee is, however, less popular than Bluetooth. Because Bluetooth is widely and easily available, it is implemented in almost all smartphones and tablets, while the ZigBee market goes around industrial applications and small home IoT applications. Green energy is also a feature of ZigBee, allowing the possibility of energy harvesting options in the stack protocol. It is designed specifically for uses with no possibility of external power and work together with extremely low power silicon devices. This way, ultra-low power communication is possible, with five or more times lower power than normal ZigBee [65]. One example case is that it is possible to use a small photovoltaic cell powered by indoor lights to send a message every minute.

Another less popular alternative is ultra wide band (UWB), which operates with pulses of bigger frequencies of the spectrum (3.1–10.6 GHz). The first consequence of this is the short-range, ideal for WSBN, but not good enough for long-distance communication in the IoT and IoMT last layers of the architecture. Ant is also an interesting alternative, with an ultra-low power design is capable of consuming less power than Bluetooth and ZigBee [64].

Table 2 summarizes these and some other common communication protocols/technologies.

Novel and emerging communication protocols are as well being developed specifically for WSBN. One popular example is using the human body as the communication medium, also called Fat-Intrabody communication (Fat-IBC). The layer of fat that exists between the skin and the muscles has very different dielectric properties than the skin and muscle. Hence, the layer of fat acts as a parallel plate waveguide, being possible to pass a radio frequency of 2.45 GHz with manageable losses, achieving low power communication between two parts of the body depending on a fat layer [66]. It should be mentioned that to implement this communication system, two electrodes need to be implemented inside the skin in contact with the fat tissue [58], and the blood vessel orientation can also affect the signal [67]. Another way to communicate using the human body is to use the body as a group of capacitors to which two electrodes are connected in contact (galvanic coupling) or separated (capacitive coupling). This way, a signal of 1–100 MHz can be transmitted for a simple and low power communication; the common name given to his method is Intra-body communication (IBC) [68,69].

The use of passive RFID tags is another energy-efficient solution, as passive RFID tags do not require a power supply to transfer data; instead, they harvest their energy using their antenna. However, the range and data transfer are very limited. Communications using this backscattering technique has been shown for a distance of 50 cm [70].

The mobile data communication, such as 4G, offers up to 300 Mbit/s and is enough for the data transfer. The new version is the 5G, and it allows for bigger data transfers of 100–900 Mbit/s. It sacrifices range, using 600–700 MHz low band, or using other mid-band with 2.5–3.7 GHz. It allows the use of high-band with 25–39 GHz achieving (Gbit/s) though. This generation is not yet widely available; still, there are already implementations for IoMT systems with it [71,72], with the possibility of complex exams and body imaging needing much data transferred.

**Table 2:** Most common wireless communication standards.

	Recent Version	Range (m)	Data Rate	Frequency	Band	Standard	Energy Consumption
**Bluetooth** [63]	5.2 (2020)	<10–500+	1–3 Mbit/s	2.402–2.480 GHz	ISM	IEEE 802.15.1	<30 mA
**Bluetooth Low energy**	-	500+	125 kbit/s-2 Mbit/s	2.400–2.4835 GHz	ISM	-	<15 mA
**ZigBee** [73]	2015	10–300+	250 Kbit/s	2.40 GHz	ISM	IEEE 802.15.4	<16 mA
**UWB** [74]	-	short	675 Mbit/s	3.1–10.6 GHz (500 MHz channels)	-	IEEE 802.15.6-2012	
**ANT** [75]	ANT+	30	60 Kbit/s	2.4 GHz	ISM	-	<60µA
**RuBee** [76]	-	20	1200 kB/s	131 kHz		IEEE 1902.1	
**Sensium** [77] (HR monitor)		>3	160 kb/s	900 MHz			<3 mA
**Zarlink** [78] (implants)	ZL70101 ZL70081 ZL70250	<2	<800 kb/s	402–405 MHz;	MICS/ ISM		<6 mA
**Z-Wave** [79,80] (Homecare)	Z-Wave Plus V2	10–100	100 Kbit/s	2.4 GHz and 900 MHz	ISM	IEEE 802.15.4	<38.8 mA (13 dB) <12.9 mA (0 dB)
**NFC** [81]	-	0.1	424 Kbit/s	13.56 MHz	ISM	ISO/IEC 18000-3	-
**RFID** [82]	-	<12 (100)	-	120–150 kHz; 13.56–928 MHz; 2.45–5.8 GHz	ISM	ISO/IEC 18000	-
**Mobile** **technology** [83]	5G	-	100–900 Mbit/s	600–700 MHz	-	-	-

## 3. Novel Forms of Conformal Bioelectronics

Smartwatches and fitness tracking solutions are already in the market for monitoring of sports activities and some basic health parameters. However, these are generally limited to specific parts of the body, such as the wrist and the chest, which limits the parameters that can be monitored. In addition, these devices lack a combination of comfort and skin contact. That is a conformable contact that is necessary for high-quality signal acquisition usually comes at the cost of reducing comfort. That is one of the motivations behind the novel field of soft and stretchable electronics that studies materials and methods for the fabrication of stretchable circuits over elastic polymers and textiles. Different forms of these have been thought of as a second augmented skin, or ultrathin electronic tattoos, [84,85,86] and other types of printed stretchable circuits. Unlike the traditional wearable with rigid elements, stretchable patches can be applied anywhere on the body, thus can be applied for a wider range of electrophysiological signals. These patches are also more comfortable and conformal to the body and can sustain the dynamic morphology of the skin. It has been as well shown that the quality of the acquired signal is better in soft and thin electronics [34], as the form factor and the softness of the electrodes contributes to better conformance of the electrodes to the skin morphology, thus resulting in a lower skin–electrode impedance, thus resulting in a higher signal to noise ratio.

Compared to smartwatches that are mostly limited to monitoring the physical activity and heart rate, the application of stretchable patches for wearable monitoring has been shown for a wide range of electrophysiological signals, including monitoring of EMG [85], ECG [87,88], EEG [85,89], respiration rate [90,91], body temperature [92,93], blood SpO_2_ saturation [94,95,96], throat, heart, and instance sounds [39,97,98], among others [99,100].

Despite these advantages, there are still several challenges to be addressed for further application of e-skin patches. This includes fabrication challenges, durability, interfacing between rigid microchips and stretchable circuits, and energy autonomy (Table 3).

### 3.1. Materials and Fabrication, Challenges and Methods for Bioelectronics Circuits

#### 3.1.1. Fabrication Challenge

**Figure 3:**
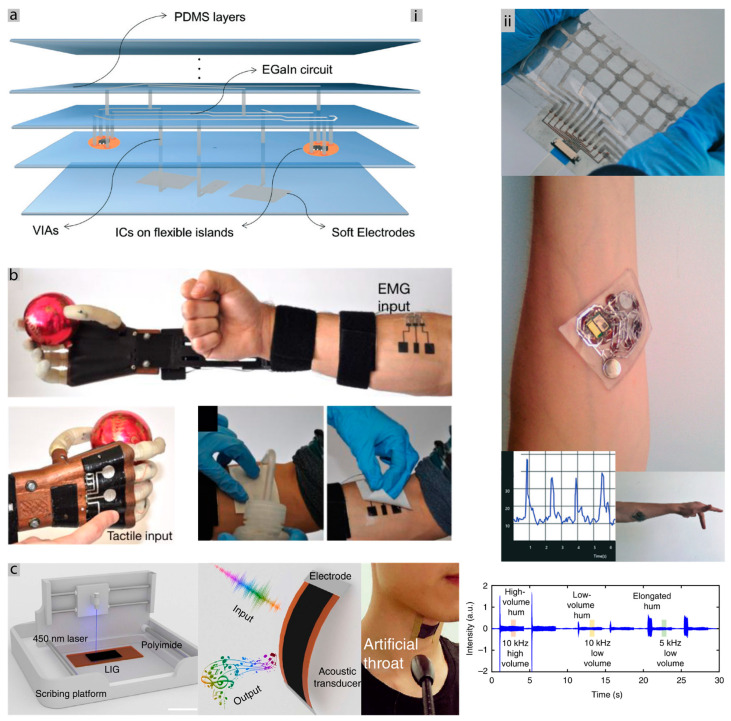
Examples of different approaches to the fabrication of bio-electronic wearables. (**a**) Multilayer stretchable patch with integrated microchips for electromyography (EMG) monitoring. (i) layer-by-layer schematics. (ii) Real applications. Adapted with permission from [108]. (**b**) ultrathin, printed electronic tattoo transferred over the forearm as an EMG input to control prosthetic hands, and as well transferred on the shield of a prosthetic hand as a tactile input. Adapted with permission from [85]. (**c**) Laser one-step induced graphene flexible sensor and transducer, capable of sound detection and sound emission. Due to the implementation of an artificial intelligence system, it has the capability of working as an artificial throat, translating hum sounds into speech. Adapted with permission from [97].

In the last decade, materials and methods for the fabrication of stretchable electronics have progressed rapidly; however, further progress is still necessary for scalable fabrication of these e-skin patches. Materials range from conductive composites, thin metal films, and liquid metals such as the eutectic gallium (EGaIn), that are patterned over elastic substrates using techniques such as laser patterning [101], lithography [102], and direct printing [103,104], high-resolution inkjet printing [105], which we covered in our recent review article [106], although, none of these methods has demonstrated scalable fabrication successfully. Some of these methods include complex steps, costly manufacturing processes and multiple times non-scalable. However, the recent advances in printed and stretchable electronics make the promise that this challenge is being surpassed [31,33,106,107]. We tackled some of the fabrication problems, presenting some methods for making complex flexible PCBs, using an infrared laser, various layers of Polydimethylsiloxane (PDMS) with EGaIn tracks are made using hole vias to communicate between them, also including rigid islands of PCB with chips Figure 3ai. An EMG prototype was built, as seen in Figure 3aii, and can detect the movement of a finger [108], while Figure 3b shows the option of electronic “tattoos” prototyping, which was used to acquire EMG signals and to control a prosthetic hand [85]. Both solutions include cheap and reproducible steps, offering many possibilities in terms of scalability. A good example of the fabrication step for these types of devices can be seen in Figure 3c, where a one-step fabrication of a graphene detector and transducer of sound was produced [97].

In the future, further development of these methods of fabrication would include a study for a reliable way to produce these types of materials in large scale and low-cost manufacturing. For example, regarding working with liquid metal materials, there exist methods for rolling [109] and one-step transfer [110]. However, these are far from perfect. They can be very limited in terms of the substrates used, and the resolution is usually not smaller than 100 μm. Moreover, even though these types of methods are very fast to produce prototypes, a method of rolling can waste much material since all the surfaces must be coated with liquid metal (very expensive material) or similar. Digital methods are the future of these approaches as they can be produced on a large scale with low wasted materials.

#### 3.1.2. Long Term Use Challenge

The second challenge is related to the long-term application of these patches. Here there are sub-challenges related to the long-term adhesion, durability of the materials and skin breathing. For instance, ultrathin electronic films or the so-called electronics tattoos conform and adhere well to the skin. They are very comfortable and imperceptible for long-term use. However, these are generally ultrathin (<10 µm thick) and fragile, being reported to be operational for a few hours [85,87]. While materials and methods for these ultrathin tattoos are advancing, other more resilient forms of multilayer stretchable electronics that are 1–3 mm thick [88,108,111] can operate for several days.

**Figure 4:**
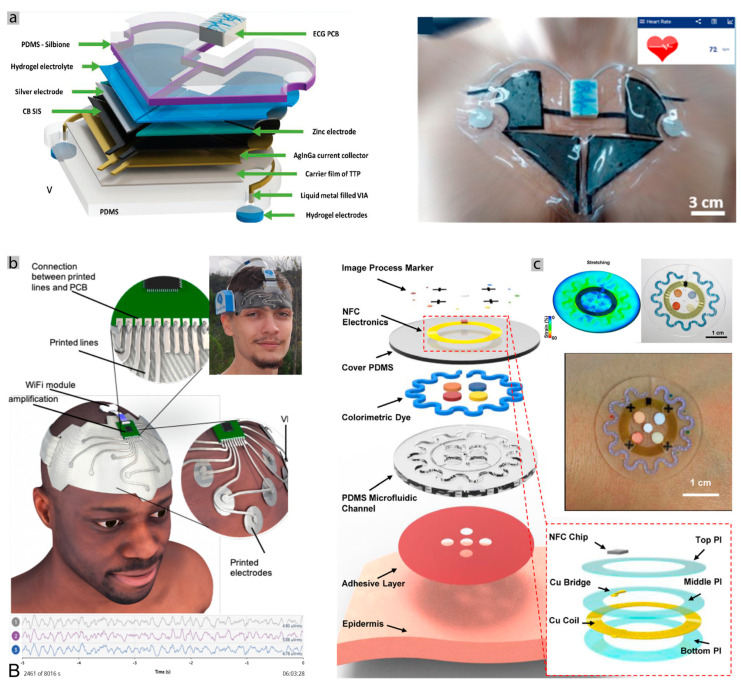
Examples of different wearables conformal to the skin. (**a**) Fully untethered electrocardiogram (ECG) patch with integrated stretchable printed Ag–Zinc battery able to work for 6 days. Adapted with permission from [88]. (**b**) Comfortable and affordable e-textile headband for electroencephalogram (EEG) acquisition. It is possible to be worn for extended periods of time, as can be seen in the EEG signal captured during sleep. Adapted with permission from [112]. (**c**) Adhesive colorimetric sensor of sweat. It has a microfluidic system for sweat capture and chemical analysis for different factors. Furthermore, it has Near Field Communication (NFC) communication without a battery. Adapted with permission from [113].

Figure 4a shows the implementation of a patch that integrates a printed silver-zinc battery that is used to power an ECG board. The patch can measure and transmit the heart rate via Bluetooth for about six days [88]. However, in this solution, the electronics circuit is built over a traditional PCB and interfaced with the stretchable printed patch. As an alternative, silicon chips can be integrated into more complex, multilayer circuits which are stretchable. 

Figure 4b shows the prototype built and tested for capturing EEG signals. This prototype was built on top of a headband with the electrodes and rigid PCB parts built-in. It can be worn for several hours, even during sleep, as can be seen in the graphic, which represents the EEG signal captured during sleep. On top of that, this solution was proven to be cheaper than other similar commercial options [112].

In terms of the issue of skin adhesion, commercially available medical adhesive films like Tegaderm are also used as a substrate for the fabrication of the biomonitoring patches.

Figure 4c shows an implementation of a colorimetric sweat sensor using a PDMS body and microfluidic chamber with an acrylic adhesive that is supposed to adhere five times better than a commercial Tegaderm adhesive while lasting for a long time [113].

For strict and reliable patient monitoring, it is argued that such systems should not be easily removable by patients. One particular case is tracking COVID 19 patients. Some works have, for instance, worked on types of semipermanent adhesion that can be taken out only at the clinic using a light with a specific wavelength [114]. Most of these materials reported allow for a good skin-adhesion, allowing for the skin to breathe, and they are biocompatible; some of them even resist water.

#### 3.1.3. Interfacing Challenge

The interfacing challenge for e-skin patches is of great importance because it includes the challenge of the interface with the skin. That is the good contact for electrodes and other electrophysiological connectors that necessitate a good signal to noise ratio to capture a useful bio-electrical signal. Furthermore, interfacing also occurs between the rigid parts of one circuit, the microchip and SMD components, and the “soft”, stretchable parts of the circuit, the interconnects.

**Figure 5:**
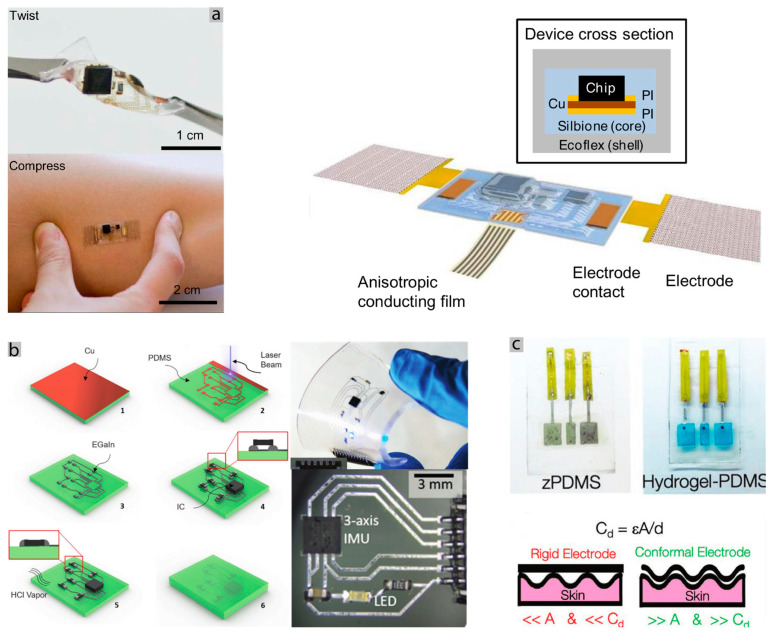
The interfacing challenge (**a**) A mechanic-acoustic detecting sensor with copper serpentine traces encapsulated in polyimide. This device is stretchable and capable of detecting sounds, specifically heart rate and murmur sounds, thanks to its skin-interfacing electrodes. Adapted with permission from [98]. (**b**) Interfacing EGaIn with rigid microchips. The process uses a copper plate carved by the laser and selective wets to EGaIn. After the microchips are placed, an HCl vapor treatment “solders” them into place. Adapted with permission from [115]. (**c**) The lack of conformity that rigid electrodes present versus soft electrodes such have silver-coated nickel anisotropic conductor (ZPDMS) and hydrogel ones. These electrodes allow for a good skin-sensor interface. Adapted with permission from [34].

Regarding the skin-device interface, the signal quality is derived from the good contact with the skin and the area available. If the electrode is not conformed to the skin, it will have worse SNR. One example of bad conformity for the skin is the commercial Ag/AgCl electrodes, which are used to acquire bio-signals. They are rigid and can become uncomfortable to use for extended periods. Figure 5c shows a study of conformity regarding electrodes, presenting a relation between the conformity with the skin and SNR. In this study, the hydrogel electrodes and tattoo ultra-thin electrodes were able to have a better SNR and adhesion than the commercial solution. Figure 5a shows an example where an Ecoflex shell system allows for good conformity to the skin for embedding an electronic mechano-acoustic sensor, with interfacing between interconnects and rigid components. While in the extremities, two reusable gold electrodes assure a good interface with the skin [98].

Interfacing rigid or soft interconnects with SMD components can be a difficult challenge if both materials do not bond to each other. One way to solve this problem, specifically when using liquid metals and rigid components, is by using multilayer stretchable circuits. Vias can be easily fabricated with laser ablation and filled with LM. This way, a flexible PCB is located in a low layer, and the soft interconnects in the layer above, as can be seen in Figure 3a [108], is not only a fabrication solution but also an interfacing one. Another different approach for interfacing with LM simply passes by placing copper wires or traces in the place to connect with the LM and protect the whole structure with an elastomer. This encapsulation will ensure the protection and the contact between both materials [116]. Anisotropic conductive films are also a viable, simple solution to rigid interface components with the interconnects [85,101]. Where all of these approaches are very interesting and relatively easy to fabricate, they do not “solder” the materials they need encapsulation. Figure 5b shows a different implementation where HCl vapor is used to effectively bond the rigid SMD structure with the EGaIn, which is already bonded to the copper traces and embedded in the PDMS [115]. Future research passes by developing bonding methods to produce scalable stretchable circuits.

Overall, the combination of the novel methods for scalable fabrication of thin but resilient multilayer stretchable circuits, and engineered bio-adhesion, is gaining enough maturity to promise the implementation of the next generation of wearable computing devices that are soft, thin, stretchable, and comfortable. Despite this, the well-known challenge of energy autonomy needs to be addressed to unlock further progress and will be discussed extensively in Section 3.2. Data security will not be discussed in this article, as it is behind its scope.

### 3.2. Energy Supply for Wearable Biomonitoring

One main challenge in the field of wearable biomonitoring is the energy autonomy of the solution. Rigid batteries such as miniature coin-sized batteries and thin-film flexible batteries are the current commercially available solutions. Yet, considering the ultimate goal of conformal and comfortable patches for long-term biomonitoring, these batteries are still bulky and not conformable. Therefore, it is desired to create solutions that are soft, stretchable, and can be integrated into these patches. This includes printed and stretchable energy storage devices, including batteries and supercapacitors, and printed, thin-film, and flexible/stretchable energy harvesting solutions, including RF antennas, photovoltaics, thermoelectric, piezoelectric, and triboelectric solutions.

**Figure 6:**
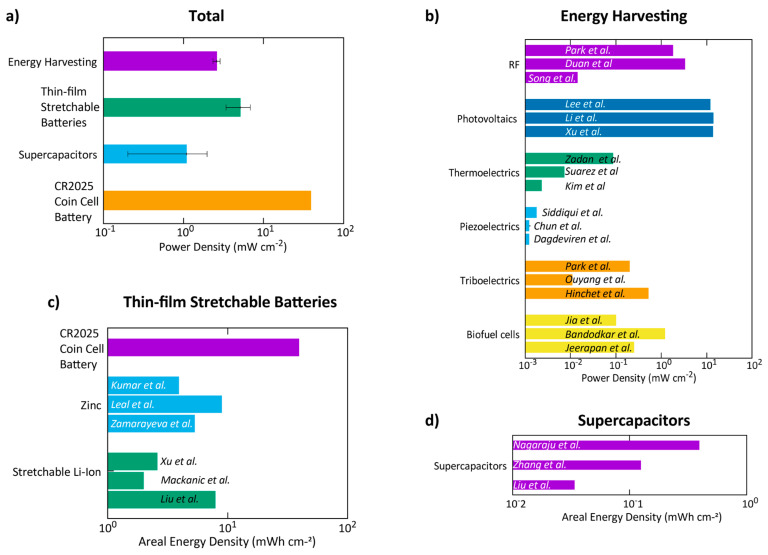
Energy storage and harvesting through thin-film or printed systems. (**a**) all the averaged energy options compared to a coin-zed battery. (**b**) Energy harvesting radio-frequency (RF): Park et al. [121], Duan et al. [122] and Song et al. [123]. Photovoltaics: Lee et al. [124], Li et al. [125] and Xu et al. [126]. Thermoelectric: Zadan et al. [127], Suarez et al. [128] and Kim et al. [129]. Piezoelectric: Siddiqui et al. [130], Chun et al. [131] and Dagdeviren et al. [132]. Triboelectric: Park et al. [133], Ouyang et al. [134] and Hinchet et al. [135]. Biofuel cells: Jia et al. [136], Bandodkar et al. [137] and Jeerapan et al. [138]. (**c**) Thin-film stretchable batteries compared to coin-size. Zinc-based: Kumar et al. [139], Leal et al. [88] and Zamarayeva et al. [140]. Stretchable Li-Ion: Xu et al. [141], Mackanic et al. [142] and Liu et al. [143]. (**d**) Wearable supercapacitors: Nagaraju et al. [144], Zhang et al. [145] and Liu et al. [146].

Figure 6 compares the areal energy and power density of some of the recent works in this area, including thin-film flexible or stretchable solutions for energy harvesting or energy storage. Figure 6a shows a comparison of all these categories with a commercial coin-cell lithium battery. Bear in mind, that for comparison, batteries were considered as doing ideal work for one hour at the maximum reported values, while supercapacitors have the power density values reported. Although energy storage and energy harvesting solutions cannot be directly compared together, this graph gives a good overall picture of each of these solutions for the sake of comparison.

Among these categories, supercapacitors offer the least power density, in the order of 1 mWh/cm^2^. However, the interest in supercapacitors is due to their rapid charging and a long number of charging/discharging cycles. The fast discharge cycles make it that the power density can have a considerable high value. Relatively, when comparing batteries and supercapacitors in terms of areal energy density gives really small values for supercapacitors (0.1 mWh/cm^2^) (Figure 6d) while batteries have an average of 4 mWh/cm^2^. Usually, supercapacitors are combined with another energy harvesting solution such as piezoelectric Nanogenerators, or antennas, to store the harvested energy and supply it to the system. In these cases, every time the supercapacitor has enough energy, it turns on the system and communicates the collected data (intermittent operation). If a continuous operation of the biomonitoring patch is required, supercapacitors can be used in combination with a battery in order to extend the energy autonomy of the system.

Stretchable batteries, on the other hand, deliver high values of areal energy density (Figure 6c), in the order of 1–10 mWh/cm^2^. While these are still almost one order of magnitude lower than commercial coin-cell batteries, these battery solutions are rapidly improving and are very promising for wearable biomonitoring solutions. The fact that these batteries are usually made by lamination of ultrathin films of stretchable polymers, and composites, make them attractive, as several battery cells can be laid over each other for achieving higher storage capacity while keeping the overall stretchability and form factor. Also, as these batteries are printed over the same polymeric/textile substrate of the wearable sensor system, they can become an integrated part of the wearable patch, thus resulting in a comfortable, light-weight and conformal solution. Among these batteries, printed Ag–Zinc batteries seem to be very promising, with values already passing the Lithium-Ion batteries. Leal et al. [88] present the best areal storage capacity value in this group of batteries. The solution with a stretchable Ag composite electrode and a zinc electrode that is fabricated over an AgInGa current collector is deposited over a tattoo paper with a hydrogel electrolyte and encapsulation of PDMS. In total, this battery can deliver close to 9.18 mWh/cm^2^ at 1.8 V, and the prototype built was able to feed a biomonitoring patch for sending the heart rate for almost 6 days. Other cases worth mentioning regarding batteries are sodium batteries, due to their non-toxicity, similar to the Ag–Zinc batteries. Guo et al. [117] presented a flexible sodium-ion battery based on a NA_0.44_MnO_2_ cathode and a nanosized NaTi_2_(PO_4_)_3_@C cathode with areal energy of 0.47 mWh/cm^2^. Also, stretchable metal–air batteries are being studied. In this type, one electrode is made of air and material for the oxygen reactions, such as carbon nanotubes. The other electrode is made of zinc lithium. Traditionally, these types of batteries were rigid and bulky. But recently, stretchable zinc–air [118,119] and lithium–air batteries have been demonstrated [120]. In theory, zinc–air batteries should have an energy density five times bigger than lithium-based ones (1086 Wh/Kg) [118], which is an impressive value considering the performance of the other types of batteries. Presently, some articles are exploring this research area; some examples reported, respectively, 5.7 Wh/L [118] and 930 Wh/kg [119]. In the end, both solutions use a rigid spring structure to achieve stretchability. The result is a nonplanar battery, although they can be made relatively thin.

While printed and stretchable batteries are still the most reliable solutions for wearable biomonitoring, methods for thin-film and wearable energy harvestings are being investigated, with the ultimate goal of battery-less solutions Figure 6b. Among them, photovoltaic solutions can achieve the best harvesting capacity while maintaining stretchable characteristics. However, dependency on light is a limitation. For an IoMT solution, with patients spending long periods indoors, can be unpractical.

Piezoelectric energy harvesting has also attracted interest, as it relies on transducing the body motion into electrical energy. In one sense, the amount of harvested energy is in the range of µW/cm^2^, which is four orders of magnitude less than photovoltaic solutions. In another sense, piezo generators can be used as a standalone, battery-less device for monitoring biomechanical signals, and their application has been shown in monitoring heart rate, respiration rate, and foot pressure map during walking [132].

Another alternative is thermoelectric energy harvesting. These are transducers that exploit the difference in temperature between two sides of materials to produce electrical energy. For instance, a patch that is placed over the skin uses the temperature gradient between the body and the air. Zadan et al. [127] demonstrated a thermoelectric generator using liquid metal embedded elastomer composites with up to 100 integrated semiconductor elements. Using this architecture, a power density of 86.6 µW/cm^2^ was achieved, using a temperature gradient of Δ60 °C. Triboelectric harvesting is based on electron transfer from contact during mechanical movements. Similar to piezo generators, these devices can be used for monitoring respiration and heart rate. Ouyang et al. [134] reported a triboelectric generator based on a sandwich of multiple layers, two triboelectric layers (PTFE and Al) would transfer surface electrons upon contact movements. Teflon and PDMS were used for encapsulation, 3D elastic sponge as a spacer, and a Kapton layer was used to incorporate a Ti keel to maintain the original shape. This module was implanted in a live pig heart with a power module regulation to generate enough power for a working pacemaker, which is about 0.377 µJ for each heartbeat. The proposed solution reached 0.495 µJ.

Even other option for energy harvesting using the human body, are biofuel cells. The basic fundament of a fuel cell is to convert the chemical energy of a certain fuel into electricity via redox reactions. A good candidate to be used as fuel is body sweat, or more specifically, the lactase present in the sweat. Bandodkar et al. [137] work used an interdigitated electrode system, using carbon-coated Au islands connected with serpentines to ensure stretchability. The anode was made of carbon nanotube-naphthoquinone (CNT-NQ), while the cathode was made of compact 3D carbon nanotube-silver oxide (CNT-Ag_2_O). The rigid islands that made the cell were implemented in a stretchable substrate, producing up to 1.2 mW/cm^2^. This means it was able to power a Bluetooth communications module. This type of cell, since it is dependent on the quantity of sweat, can also work as a self-powered sweat sensor [138]. Biofuel cells can also be used as batteries. Made with organic materials using enzyme or glucose reactions, these batteries are compatible with the body, with the possibility to be used as an implantable device. They can be made with enzyme-modified conductive textiles electrodes as reported by Ogawa et al. [147] for a power density of 0.2 mW/cm^2^. Or with metallic cotton fibers, with glucose in the anode reported by Kwon et al. [148], which claims a power density of 3.7 mW/cm^2^.

**Figure 7:**
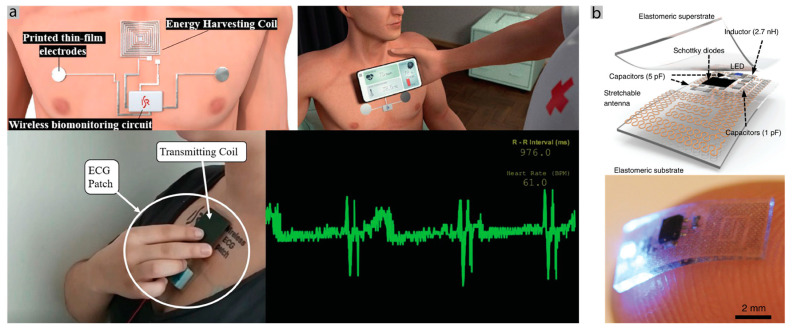
Energy harvesting devices. (**a**) Fully wireless and battery-less ECG patch, powered by near field radio frequency energy harvesting. Adapted with permission from [87]. (**b**) Miniaturized implantable optogenetics system, of rigid, interconnects Ti/Au in a serpentine shape. It has a stretchable antenna being capable of energy harvesting by radio waves. Adapted with permission from [121].

Radio-frequency harvesting uses the radio waves that surround us as the source of electric power. RF harvesting can exploit the radio waves scattered for other purposes, or specific waves, that are produced by a transmitter for wireless energy transfer. The radio-frequency energy should be captured by the harvesting device and then converted to electrical power. So, the traditional method to proceed to implement an RF harvesting system is to use a designed antenna and rectifying circuit (rectenna). As an example, Song et al. [123] used a cross dipole antenna on top of an FR4 substrate and the rectifying circuit on a PCB, achieving 70% conversion efficiency at 2.15 GHz with an incident power of 1 mW. Another possibility to achieve the harvesting of the radio frequencies is to use a metamaterial rectifying surface (MRS). These are man-made materials that manipulate the effective permittivity and the permeability of the material, thus getting the desired characteristics for better efficiency. Duan et al. [122] implement this with a three-layer PCB. Metamaterial particles in the front, a metal ground in the middle and the rectifying circuit in the back. With the optimal size of the metamaterial particles, they achieve an impedance close to free space, obtaining full capture of electromagnetic waves with minimum reflection. This design is capable of a harvesting efficiency of 66.9% at 2.45 GHz when using an incident power density of 5 mW/cm^2^. Both these works used rigid boards. Meanwhile, Park et al. [121] explore the introduction of a stretchable RF harvesting mechanism used to turn on an LED implanted in a mouse. They made a stretchable antenna using a serpentine geometry and could significantly reduce the dimensions of the system 100-fold, compared to normal antennas. It used a 0.3 × 0.3 cm antenna, making it ideal for body applications as shown implanting it in the mouse (Figure 7b).

#### Energy Consumption

In order to understand the suitability of the energy storage and harvesting solutions for wearable biomonitoring, it is important to evaluate the consumption of electronic components for wearable biomonitoring, including sensing devices (e.g., body temperature, heart rate, and pulse oximeter sensors), processing microchips, and communication chips. Table 4, presents some typical values of the required operating voltage and current for a few solid-state electronic components that could be used to implement wearable solutions. It should be noted that due to the interest in low-consumption and wearable devices, a novel generation of low power electronics is emerging with deep sleep modes or equivalent. In this mode, these devices can consume only a few µA. Looking into Table 2, these devices operate with a voltage ranging from 1–5 V and power consumption of up to 16 mA. It can be seen that data communication is the most power-hungry part, with a required current of up to 16 mA, while other sensors require less than 1 mA for continuous operation and processing power requires between 2 and 5 mA (depending on frequency). On the other hand, Bluetooth communication can be optimized to switch between an active and standby mode to reduce power consumption. Therefore, the frequency of data acquisition becomes a very important factor. For instance, a heart-rate monitor can communicate the data every second, while full monitoring of ECG requires sending data at a frequency of over 30 Hz. In practice, new wearable solutions, such as Movesense [149] that monitor the heart-rate, ECG, and temperature, has a continuous power consumption of less than 7 mW. This suggests that some of the energy harvesting solutions, such as RF-based antennas, are close to delivering the required power of wearable solutions. Rapid advances in low-consumption communication technologies, and improved efficiency of RF antennas, seem to make the promise for the next generation of battery-less biomonitoring patches, although there is a need for an RF transmitter nearby. For the current technology, however, it seems that a combination of a printed stretchable battery and an energy harvesting device is the most feasible solution for long-term wearable biomonitoring.

Figure 4a represents an example of a printed battery over a stretchable patch. Even though it is, in fact, two batteries in series and covers a big part of the chest, it can communicate the heart rate for six days without any harvesting device. Also, the optimization of power consumption is an important factor to get the maximum of the energy budget, which is a subject of some research works that report optimization algorithms that increase energy autonomy 2.4 times, compared to the manual optimization methods [150]. In an opposite approach, Figure 7a represents a fully wireless electronic tattoo for ECG monitoring that harvests its energy through near field electromagnetic coupling. A coil antenna was designed and printed using conductive and stretchable ink over a temporary tattoo paper, which was then transferred to the human body. As proof of concept, the circuit of the ECG was powered by this energy harvesting solution [87]. This prototype is useful to show the scalability of the fabrication process because of dimension, fabrication and successful application while using a good conformable substrate and an energy harvesting solution.

## 4. Sensing Architecture for Covid-19 Patients

At the end of the year 2019, a severe acute respiratory syndrome coronavirus 2 (SARS-CoV-2), commonly referred to as Covid-19 [151], appeared in the Hubei province in China, which lead to a global epidemic due to the rapid increase of the number of people infected with the virus during the year 2020. According to reports, most of the infected patients do not need intensive care. Only 9–11% of patients need intensive care [2]. Considering that countries have very few beds available for the intensive care unit (ICU), for example, Italy has only 5200 beds for intensive care [2]. Therefore, most countries were forced to employ a “domiciliary hospitalization” model, in which only patients with a severe respiratory problem were hospitalized [149]. The rest of the patients were treated at their home while receiving medical advice through the phone. However, deciding on which of the patients hospitalize was largely based on how the patients feel, and not a medical measurement. In addition, due to the lack of beds in ICU, doctors had to make difficult decisions on the admission of patients in ICU, based on their life expectancy. In all these cases, low-cost wearable tools for long-term monitoring of patients could bring several advantages. First, for patients hospitalized at home, these devices could provide medically relevant measurements, generate indicators, and detect emergency cases. Second, it can provide a decision-making support tool, to help making decision based on clinically relevant measures. This includes which patients to hospitalize, and from those, how to decide which patients should move to ICU. Third, gathering big data from a large number of patients, could result in discovery of digital biomarkers, and algorithms that can combine multiple data sources, such as thermal, mechanical, and bioelectrical data to classify infected patients. As an example, the correlation between other types of Corona viruses, and ECG data were studied over 20 years ago [152]. Fourth, a wearable biomonitoring tool can be used for tracking the patient location, to ensure confinement rules, and to build dynamic COVID risk maps. Nevertheless, this last application requires addressing the privacy issues, since locating patient location at all times can be a sensitive matter [153].

### 4.1. How to Monitor a Covid-19 Patient

**Figure 8:**
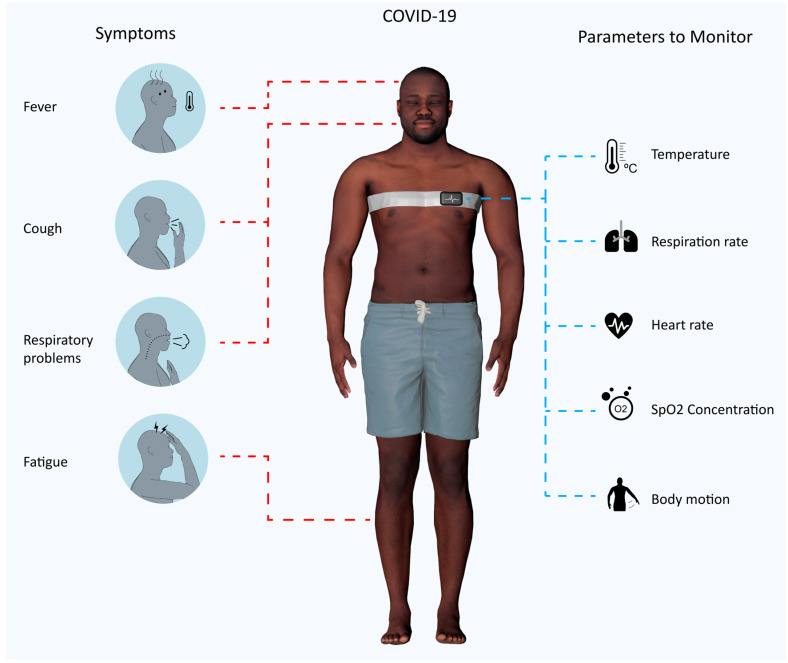
The most common Covid-19 symptoms and the proposed monitoring parameters.

Covid-19 is a severe acute respiratory syndrome, and according to Grant et al. [154], the most common symptoms are fever, coughs, new coughs and dry coughs, and fatigue. As such, it is important to monitor respiration rate for respiratory problems, blood oxygenation and temperature. Blood oxygenation is a measure that is used to evaluate the efficiency of the respiratory system to deliver enough oxygen to the blood and is one of the measures that is used currently to evaluate the severity of each case and can also give input as the fatigue. In addition to this, the heart-rate or ECG signals, and body temperature, are among parameters that are monitored in order to evaluate the general well-being of the patients, as well as a precaution, since patients can develop various cardiac problems, like arrhythmia, myocarditis, heart failure and others [155]. Studies that monitor heart rate instead of a complete ECG for coronary problems exist [156], and heart rate was often considered as important data to be collected in other outbreaks [157,158]. Body motion can be monitored as well for detection of collapses and fusion with other sensors. For instance, SpO_2_ measurement is very sensitive and should be measured when the body is not moving. Considering the most common conditions, we propose a wearable chest band architecture that collects all these biosignals (oxygen level, ECG, heart rate, body temperature, and body motion) in Figure 8.

Heart rate and Electrocardiogram: The most common way of measuring the heart rate is by measuring the electrical signal on the patient’s chest, that is, performing an electrocardiogram (ECG). ECG contains the entire electrical signal produced by the heart, from which the heart rate can be, as well, obtained. ECG can be performed with at least two electrodes [159]. Although, a one lead ECG using one layout with 3 electrodes close to each other in a neckband was shown [160]. When considering a wearable solution, the skin-interfacing electrode is the most important component of the system. To obtain a reliable and high-quality signal, the electrode should establish a reliable mechanical and electrical connection with the skin. That means that the electrode should either adhere or be pushed into contact with the skin, and it should establish a low skin-electrode impedance, to obtain a high SNR. Electrodes are divided into two categories: wet electrodes and dry electrodes [161,162]. There exist some examples of textile electrodes based on yarn coated with silver [163], thread coated in silver [164] and nanoweb coated with silver nanowires [165]. Other types of electrodes can be made using conductive pastes that can be screen-printed on fabric [166], or conductive inks of gold [167], silver ink that permeates the fabric [168] and graphene ink also applied to a fabric [169]. The overall objective is to have a reliable signal while keeping the solution wearable and comfortable. Existing literature compared different electrodes in terms of reliability of the contact, quality of the signal, or even wash capability [170,171]. Recently, the use of tough, conductive, and stretchable hydrogels as electrodes received increasing attention. In this work [34], we compared hydrogel electrodes with 6 other types of electrodes, including commercial Ag/AgCl, medical-grade stainless steel, printed tattoo electrodes, silver-PDMS, carbon-PDMS, and silver-coated nickel anisotropic conductor (zPDMS). We showed that hydrogels, present the best performance among all the others, including the lowest skin–electrode impedance, and highest SNR value, better than gold-standard Ag/AgCl electrodes. In addition, noncontact capacitive coupling electrodes has been studied [172] lately. These are being investigated as a noncontact solution, but usually suffer from a low SNR. Even with acoustic solutions, it is possible to obtain the sound related to heart valve movement and therefore calculate the heart rate [98] (Figure 5a).

It is worth mentioning that even though low power ECGs are available [173], ECG communication is generally power-hungry. In contrast to the heart rate, or respiration rate, that require sending the data at ~1 Hz, a full ECG requires digitizing the ECG curves and send data at a much higher rate.

**Figure 9:**
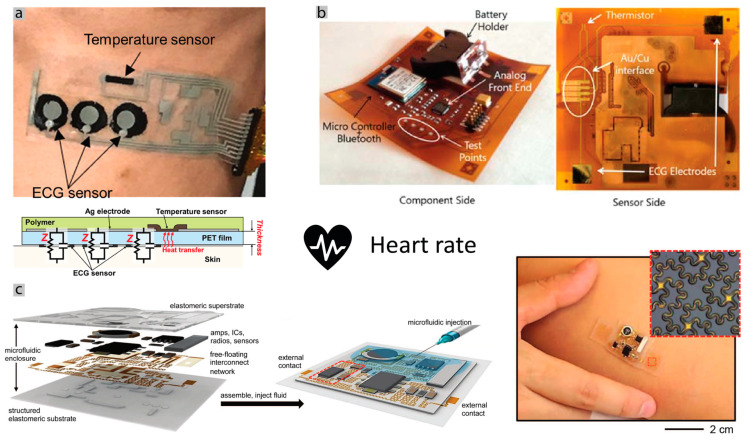
Examples of sensors used for ECG and heart rate monitoring. (**a**) ECG and temperature sensor solution based on stretchable PDMS with PEIE making gel-less electrodes. Adapted with permission from [174]. (**b**) Flexible ECG board with inkjet-printed electrodes on a Kapton substrate. Adapted with permission from [175]. (**c**) ECG sensor solution based on free-floating rigid interconnects in serpentine shape, inside of a microfluidic enclosure. Adapted with permission from [176].

Some good examples of fully functional wearable ECG patches are Figure 9a, which demonstrates an example of a thin-film ECG patch over a PET substrate and carbon nanotubes electrodes [174]. Figure 9c shows an ECG with free-floating rigid interconnects. Figure 9b which is implemented as a double layer flexible PCB over Kapton [175].

**Figure 10:**
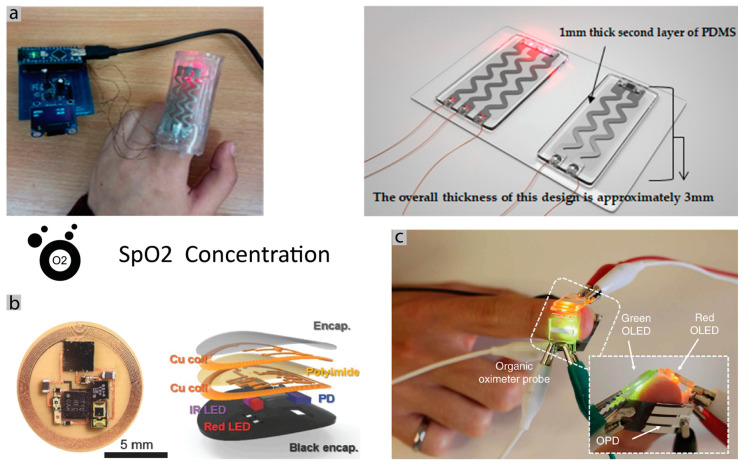
Oximeter sensors. (**a**) Oximeter sensor consisting of printed silver interfaces, on top of flexible PDMS, and connecting the LEDs and PD. It has stretchable possibilities due to geometry. Adapted with permission from [94]. (**b**) Small oximeter with copper traces and coils on top of a polyimide substrate inside a black encapsulation reduces exterior optic interferences. It has NFC communications using energy harvesting from RF waves. Adapted with permission from [46]. (**c**) Oximeter made with organic LEDs and organic PD on a plastic substrate in ring shape Adapted with permission from [95].

Oxygen saturation in the blood: To measure the oxygen saturation in the blood, normally a simple oximeter sensor can be used, based on the optical principle of emitting two different wavelengths of light into the body and capture the reflection with a photodiode. This is a simple, non-invasive method of measuring the peripheral oxygenation (SpO_2_) and is based on the different reflection of the blood hemoglobin, depending on the amount of oxygen present [177]. The preferred location for SpO_2_ measurement is the wrist, fingertip or earlobe, and there are, already, very energy-efficient systems, consuming low amounts of power [178]. Measuring oxygen saturation in the blood on the chest can be difficult because of the thickness of the skin, and consequently, the quality of the captured signal. Some works have studied this problem and proposed different architecture with the same working principle of the sensors [179,180,181]. However, due to the difference in skin thickness, these sensors use more LEDs and photodiodes located strategically to capture a good signal that is of the same quality as the finger located. Plus, these sensors have the advantage of capturing the information of the blood in the heart region (proximal), which can be advantageous as the finger location is a distal zone, and far away from the heart. One example of a flexible pulse oximeter is shown in Figure 10a. In Figure 10c, a compact design is achieved by using organic LEDs and organic photodiodes in a flexible substrate [94,95]. One interesting aspect of novel oximeters chips is that they can measure heart rate as well, performing a PPG (photoplethysmogram) that is used to detect blood volume changes in the microvascular bed of tissue, which is related to the cardiac cycle. Furthermore, the possibility of measuring the blood oxygenation by oximeter is also available within body rigid materials like nails and teeth, represented in Figure 10b. Giving the possibility of a small size (smaller than a nail) and battery-less sensor [46].

Respiration rate: Different methods have been used to measure the respiration rate. The most common methods are flow-measurement methods that use sensors located near the mouth and nose [182]. These sensors can measure air temperature, pressure, humidity and CO_2_ concentrations [91,183], as well, calculate the respiration rate. These are widespread sensors but not a practical wearable solution. Acoustic sensors can also be used to detect the respiration from sounds produced by lung movements [184]. While these can be made wearable, acoustic measurement is always prone to environmental noises [185].

**Figure 11:**
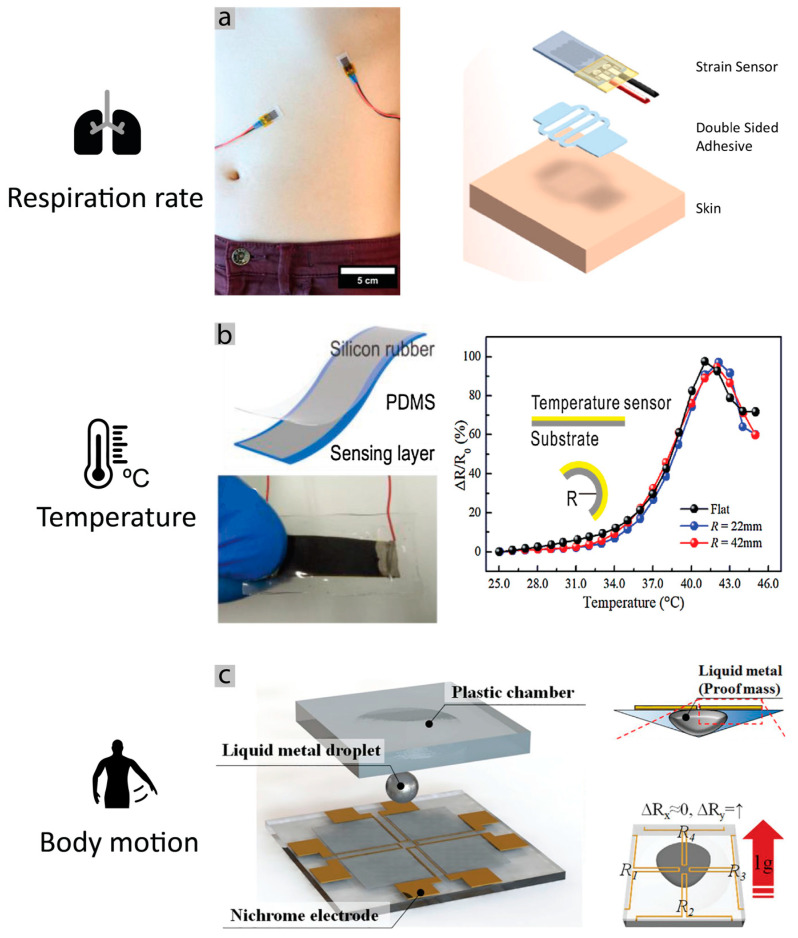
Respiration rate, temperature and motion sensors. (**a**) Respiration rate sensor using two small perpendicular strain sensors on the abdomen. Adapted with permission from [90]. (**b**) High resolution flexible small temperature sensor, based on Poly(ethylene oxide) (PEO) and graphite material, encapsulated with PDMS and silicon rubber. Adapted with permission from [93] (on the right, the performance upon bending). (**c**) Dual-axis accelerometer using a liquid metal ball as a proof mass, moving inside an encapsulation. Adapted with permission from [100].

Therefore, some works studied the detection of respiration rate, using wearable sensors for detection of mechanical movement. This includes resistive or capacitive strain sensors that are stretched due to respiration, thus resulting in the change of the resistance or capacitance value. Resistive sensors can be implemented by deposition and patterning of conductive materials over polymers or textiles [186] or by knitting conductive threads [187]. Capacitive sensors generally implement a parallel-plate capacitor, using two textile electrodes on opposite sides of the textile or a planar interdigitated capacitor, and analyze the capacitance changes [182]. Other interesting methods worth mentioning are optical fiber sensors around the torso, which change their transmittance with the expansion [187]. Piezoelectric [188] and triboelectric [189] belong to the last class of sensors that produces electrical signals with movement. In addition, sensor patches with strain gauges have been shown, using carbon nanotubes as electrodes [190]. Recent work demonstrated a strain sensors patch, able to detect respiration rate and air volume while assuring mobility of the patients [90] (Figure 11a). The system is composed of two strain sensors placed perpendicular to each other, on the abdomen and the ribcage. The sensor itself is made of a piezoresistive sensor on a silicon substrate [191]. The sensors of 21 mm by 10 mm and thickness of 0.5 mm and their positions allow for a low interference detection of expansion and contraction of the torso. Overall, utilization of resistive strain gauges seems a reliable option for integration into an e-textile chest-band for estimation of respiration rate.

Body temperature: Body temperature is relatively simpler to implement compared to previous sensors, as there exist several different technologies and microchips for temperature measuring. Flexible temperature sensors have already been demonstrated in some works [92]. Measuring the temperature can be achieved simply by using thermistors, materials, which the resistance varies in a known way as the temperature varies. In addition, photonic temperature sensors have been shown [192] as very precise measuring systems, but they usually need more complex electronics. In order to implement thermistors into flexible and stretchable patches, researchers have demonstrated various solutions, including laser-patterned nickel oxide thin films that can be implemented on an “artificial skin” [193], Graphite-filled polyethylene sensors, a PTC that exhibits 0.1 °C of accuracy temperature measure [93] (Figure 11b), graphene sensor with a zig-zag design, implemented as a thermistor that can be stretched to 50% strain while maintaining operability [194], and organic semiconductor thermistor over polyester [195]. In addition, some graphene sensors have been demonstrated that change their resistance with strain [196] or airflow [197]. While promising, we believe the numerous silicon chip solutions that are commercially available are the most reliable ones as these are miniaturized, low cost, and often integrate the communication protocol such as I^2^C, or SPI, which facilitates their integration with the central microcontroller.

Body motion: The human motion can be monitored easily using an advanced accelerometer, gyroscopes, or integrated inertial measurement units (IMU). Traditional electronic uses accelerometers extremely compact based on the cantilever principle, in which a proof mass is suspended by a spring mechanism that detects stress upon movements on the proof mass [198]. Figure 11c shows the principle using a liquid metal drop. Nevertheless, the low-cost, miniaturized IMU are usually equipped with an integrated ADC and I2C or SPI communication, making it easy to integrate them into the wearable chest-band architecture.

### 4.2. An IoMT System Dedicated to Covid-19 Monitoring

**Figure 12:**
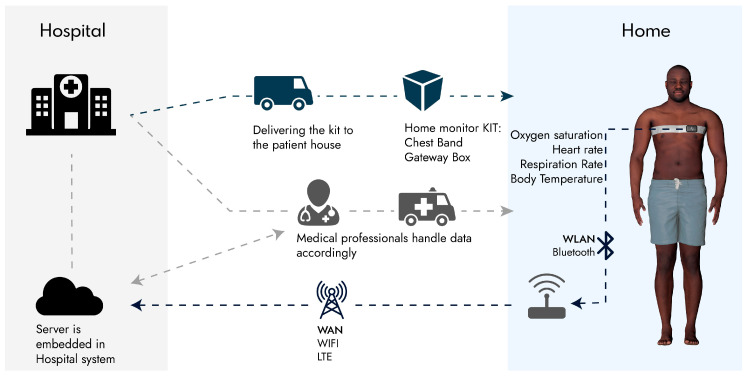
A proposed implementation for the IoMT system adapted to the pandemic caused by Covid-19.

Based on the existing biomonitoring technologies, new wearable chips, and our previous works on wearable biomonitoring, we propose an architecture for monitoring Covid-19 patients. The proposed solution is based on the general structure of IoMT previously presented. The general hardware system is composed of a wearable biomonitoring chest band, and a smart box as a gateway to obtain data from the wearable belt, process it and communicate the processed data to a central cloud belonging to the hospital. Figure 12 shows the proposal architecture, where the patient will get a kit box with the chest band and smart box at home or picks it up at a special Covid-19 zone in the hospital. The patient will set up the gateway box to a power outlet and wear the chest band at all times. The information captured by the device 24 h per day will be transmitted via Bluetooth to the gateway box. For easy implementation, this box is only responsible for data transfer and basic information display for which it can be based on a commercial board like Raspberry Pi. The gateway connects to WIFI if available or a mobile data network to send the data to the hospital-owned cloud, which will be accessible to health professionals. Healthcare professionals will be able to deploy automatic/manual emergency measures based on this information, i.e., if the patient’s symptoms aggravate or he collapses, and the box is not transmitting data, or even confinement measures. Given that user data are also important, the user should always have access to his data, so he can also ask for help in case of any doubt.

**Figure 13:**
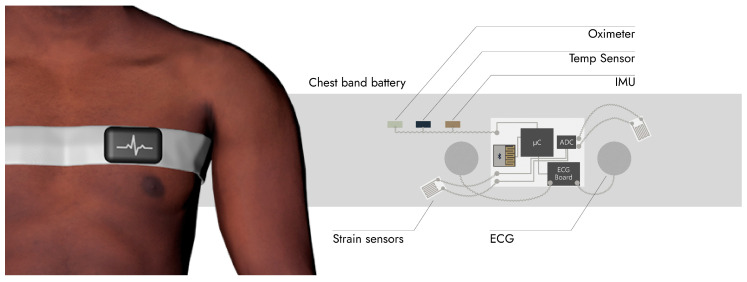
Schematics of the chest and circuit with the sensor implementation, the main chip would be the Bluetooth and μC board. With ECG board, and the multiple sensors included in the chest band, which has a battery integrated.

The proposed chest-band structure is shown in Figure 13, composed of a central processing board, an e-textile chest-band, and distributed sensors. The central processing board is composed of a microcontroller, a Bluetooth low energy chip, and a biopotential acquisition and processing chip for ECG signals with integrated ADC and amplifiers. The e-textile contains the ECG electrodes and interconnects to transmit the ECG data to the central board. It also contains strain gauges that are printed/patterned over the textile using conductive ink. Other sensors, i.e., pulse oximeter and temperature sensor, requires skin contact and should be implemented on the other side of the belt while communicating with the central board through digital protocols, such as I^2^C. Finally, the IMU can be implemented as well on the central board. However, if implemented near the temperature sensor and pulse oximeter, it is beneficial, as the motion data can be fused with the oximeter and temperature sensor so that the reliability of the measurements are weighted based on the stability of the sensors. This is because both temperature and oximeter sensors are very sensitive to changes in the contact of the sensor with the skin due to the motion, and thus a reliable measurement is usually obtained when the sensors are not moving.

A range of research and commercial wearable sensors are now available for remote patient monitoring [199]. One example of this type of fitness solution adaptation is the “Whoop activity tracker”, used to detect anomalies in respiration rate and detect COVID infection up to two days prior to symptoms for 20% of cases and up to three days after symptoms on 80% of cases. Proving how important it is to monitor the vital signs since most people are normally diagnosed around day seven after symptoms, giving this approach and advantage of four days [200]. One example of a particular sensor is a small temperature monitor for extended periods of use, and now getting used on patients of Covid-19 for a constant monitor in hospitals and at their own home, as is the case of Temp Pal (iWeeCare) [200]. Another solution is the throat patch [43], in which a small soft sensor with an accelerometer is put over the intrathoracic cavity for measuring temperature, heart rate, respiration, and cough sounds.

## 5. Conclusions

In this article, we reviewed recent advances and existing challenges of wearable biomonitoring for domiciliary hospitalization. The general structure of IoMT systems was presented, including the parameters that can be measured using bioelectrical, mechanical, acoustics, and optics sensors, as well as protocols for wireless communication of the collected data. We discussed advances in the fabrication of stretchable electronics as a step toward the implementation of wearable patches for long-term biomonitoring. We discussed, as well, the general implementation of an IoMT system for domiciliary hospitalization. As one of the existing challenges against wearable biomonitoring is related to the energy autonomy of the wearable sensors, we discussed existing commercial solutions and current research to address this issue. This included printed and stretchable energy storage devices, i.e., batteries and supercapacitors, as well as solutions for harvesting the energy through RF antennas, piezoelectric, thermoelectric, triboelectric and photovoltaic, harvesters that are implemented as thin-film and printed solutions. Finally, we discuss a possible architecture for the case of COVID 19 patients that includes a wearable chest band for data collection and a smart gateway for data transmission, with integrated sensors for monitoring ECG, heart rate, respiration rate, blood oxygen saturation, body temperature, and motions.

## Figures and Tables

**Table 1:** Types of Bio-signals, and most common tests.

Biopotential	Bioimpedance	Conductance	Acoustics	Optical	Others
Muscles (EMG)	Emotions	GSR	Voice	SpO_2_ Concentration	Temperature
Heart (ECG)	Body fat		Food intake	Pigmentation changes	Mechanical (myography)
Brain (EEG)			Digestive System		Chemical (e.g., sweat)
Eyes (EOG)			Coughs		
			Heart murmurs		

**Table 3:** Current challenges and research focus for e-skin patches.

E-Skin Patches, Current Challenges and Research Focus				
Fabrication	Long Term Use/Reliability	Interfacing	Energy Autonomy	User Data Security
Scalability	Biocompatibility	Soft/rigid interface	Energy harvest	Protocol security
Cost	Durability of materials	Microchip integration	Energy storage	Data privacy
Production time	Adhesion (removable/semipermanent)	Skin-interfacing	Power consumption	
	Skin breathing			
	Water-resistance/ re-application			

**Table 4:** Energy consumption for typical solid-state parts of the prototype.

Solid State Parts	Voltage (V)	Typical Current (µA)	Standby/Sleep Current (µA)	Example
**Oximeter**	1.8/3.3	600	0.7	MAX30102
**Temperature Sensor**	1.9–3.6	90	0.06	Si7050
**Accelerometer**	1.71–3.6	2	0.5	LIS2DH
**ECG Board**	1.1–2	100	0.73	MAX30003
**Communication**	1.71–5.5	15,600 Tx, 16,400 Rx	1.3 Deep Sleep 0.150 Hibernate 0.06 Stop	CYBLE-014008-00: EZ-BLE
**µC**	1.71–5.5	2500 at 6 MHz	1.3 Deep Sleep 0.150 Hibernate 0.06 Stop	CYBLE-014008-00: EZ-BLE
